# Supplementary material for: Content-rich biological network constructed by mining PubMed abstracts
Source: BMC Bioinformatics. 2004 Oct 8;5:147. doi: 10.1186/1471-2105-5-147 (PMC528731; doi:10.1186/1471-2105-5-147)
Supplement: Additional File 2 — The original results of the above study (non-essential files are deleted to keep the file size under the limit set by BMC bioinformatics). [file 1471-2105-5-147-S2.bz2 › chilibotAdditionalFile2/dip10/208ID8846021E708/html/ITGA1_ITGB1.html]

 


 **ITGA1** and **ITGB1** 
  
Found 245 abstracts in PubMed, retrieved 10.  
 

 What does Google say? 
 PDF only 
| .edu only 

---

**Interactive relationship** (e.g. stimulation, inhibition, etc)

**Non-interactive relationship** (e.g. studied together, co-existance, homology, etc.)

- Flow cytometry was used to evaluate the expression rate of VLA  [ **ITGA1** ]  4 CD49d CD29  [ **ITGB1** ]  and VLA  [ **ITGA1** ]  5 CD49e CD29  [ **ITGB1** ]  and beta1 integrin activation ability in the transfected K562 cells.  Ref: 12844402 Zhongguo Shi Yan Xue Ye Xue Za Zhi, 2003
- ATG also contained functional antibodies to the beta1 integrin CD49d CD29  [ **ITGB1** ]  VLA  [ **ITGA1** ]  4, the alpha4beta7 integrin, CD50, CD54, and CD102 but not to CD62L.  Ref: 12640305 Transplantation, 2003
- Data obtained are presented in concurrence with Clusters of Thymic Epithelial Staining CTES classification for various monoclonal antibodies recognizing CD antigens CD1, CD1a, CD6, CD9, CD14, CD16, CD29  [ **ITGB1** ] , CD30, CD32, CD44, CD45RB, CD47, CD48, CD49a  [ **ITGA1** ] , CD49b, CD49c, CD49d, CD49e, CD49f, CD51, CD53, CD54, CD56, CD57, CD63, CD85, CD95, CD98, CD102, CD103, CD106, CD109, CD146, CD147, CD148, CD151, CD152, CD158a, CD158b, CD164, CD165, CD166 and for monoclonal antibodies 1B1 G7, A4, BD46, BLTZ, HP1C5, IND.64, M72, WU947 whose specifities are not yet defined.  Ref: 12725440 Ann Anat, 2003
- We found that T h 1 cells derived from ovalbumin OVA specific TCR transgenic DO11.10 mice showed significantly higher levels of VLA  [ **ITGA1** ]  2 CD49b CD29  [ **ITGB1** ]  expression than T h 2 cells.  Ref: 12750354 Int Immunol, 2003
- Furthermore, eosinophil recruitment and activation appears to be mediated by a combination of VLA  [ **ITGA1** ]  4 CD49d CD29  [ **ITGB1** ]  and LFA 1 CD18 CD11a.  Ref: 12877819 Pulm Pharmacol Ther, 2003
- The data show that in the allergic rabbit model of asthma, VLA  [ **ITGA1** ]  4 CD49d CD29  [ **ITGB1** ]  only, is involved in the acute bronchoconstriction, suggesting an involvement of mast cell degranulation.  Ref: 12877819 Pulm Pharmacol Ther, 2003
